# Nutritional Status of Adult People Living with HIV: A Narrative Review

**DOI:** 10.3390/diseases13020056

**Published:** 2025-02-14

**Authors:** Stella Proikaki, Nikolaos Georgiadis, Theodoros N. Sergentanis, Eleni Kornarou, Tonia Vassilakou

**Affiliations:** MSc in Public Health, Department of Public Health Policy, University of West Attica, 11521 Athens, Greece; nikgeorgiadis@uniwa.gr (N.G.); tsergentanis@uniwa.gr (T.N.S.); ekornarou@uniwa.gr (E.K.)

**Keywords:** HIV infection, malnutrition, HIV-associated wasting, nutrition care

## Abstract

Background: The interaction between HIV infection, nutrition and immune system functioning is intricate, leading, in many cases, to a cycle of poor health outcomes. Despite the widespread use of highly active antiretroviral therapy (HAART) since the late 1990s and the concomitant increase in the life expectancy of people living with HIV (PLHIV), malnutrition and HIV-associated wasting continue to pose significant challenges, particularly in developing countries. Additionally, metabolic adverse effects associated with HAART, such as alterations in bone and lipid metabolism, as well as the impact on cardiovascular health, add further complexity to patient care. Methods: We conducted a comprehensive literature review of relevant studies involving adults diagnosed with HIV. The studies, published between 2000 and 2023, were identified using the Medline/PubMed, Scopus and Google Scholar databases. Results: Accumulating evidence in the literature indicates that careful monitoring and appropriate nutritional interventions can significantly enhance clinical outcomes in malnourished HIV-positive persons. The importance of addressing the prevalent deficiencies in certain micronutrients discussed in many of the studies is clearly underlined. However, challenges remain, particularly in low-income settings, where limited resources and infrastructure can impede effective implementation. Conclusions: There are critical research gaps with regard to the interaction between ART and nutrition, as well as the development of tailored nutritional approaches that aim to improve patient outcomes. Future research directions and policy strategies should focus on the development of sustainable programmes aimed at enhancing the quality of life for PLHIV.

## 1. Introduction

In 2023, it was estimated that 39.9 million people were living with HIV (PLHIV) worldwide, with 38.6 million being adults [1]. Nutrition plays a crucial role in bolstering the immune system, particularly in PLHIV, by helping to fend off infections, slow down HIV disease progression, and enhance quality of life and overall survival [2,3].

The extensive use of HAART since the late 1990s has contributed to the reduction in HIV-associated morbidity and mortality risk, transforming HIV infection from an almost certainly fatal disease to a chronic one [4]. Despite the significant increase in life expectancy of PLHIV, involuntary weight loss and malnutrition remain challenging issues in any stage of HIV infection [5,6]. Specifically, poor nutritional status, characterized by inadequate consumption and utilization of nutrients, is closely intertwined with both the worsening of clinical status in people with HIV and the acceleration of progression to AIDS [7]. Conversely, better nutritional status in PLHIV on antiretroviral therapy (ART) may improve the effectiveness of the antiretroviral drugs, enhance adherence to treatment, and minimize ART-related side effects, thus reducing the long-term metabolic complications of ART use (dyslipidemia, insulin resistance and obesity) [8,9]. Weight loss is a prevalent issue among HIV-positive individuals, which is often attributed to various factors such as inadequate nutrient intake, increased nutritional requirements, malabsorption, and modified metabolism, all contributing to a negative energy balance [6].

HIV-associated wasting syndrome is a severe form of malnutrition, defined as an involuntary weight loss of greater than 10% with associated fatigue, fever, and diarrhea that cannot be explained by other causes [10]. The intertwining relationship between malnutrition and HIV infection creates a vicious cycle, leading to detrimental effects on the immune system [11]. Consequently, this increases susceptibility to opportunistic infections and gastrointestinal disturbances, thereby impairing nutrient absorption [6]. Addressing these challenges necessitates the integration of nutritional interventions into comprehensive HIV care and treatment plans, including nutritional education and supplementation [12,13]. It is well known that access to health services is also inadequate in many settings, especially in low-income countries; thus, dietary interventions, particularly in resource-limited settings, are crucial to improve caloric intake and enhance the quality of life of PLHIV [14].

The present narrative review aims to provide a comprehensive overview of nutrition-related concerns and metabolic changes among PLHIV, while also summarizing interventions designed to address these issues, drawing from observational and nutrition intervention studies. Furthermore, this review aims to critically examine the literature concerning micronutrient supplementation studies, emphasizing their safety and efficacy for this particular population.

## 2. Materials and Methods

We conducted an electronic search of the international literature spanning from 2000 to 2023 across three databases: PubMeD/MEDLINE^®^, Scopus and Google Scholar. The aim was to identify articles pertinent to the review’s topic, including observational studies, randomized controlled trials, narrative and systematic reviews, and meta-analyses, published during the period 2000–2023. Our search strategy utilized a combination of keywords and medical subject headings (MeSH) including “HIV infection”, “HIV-associated wasting”, “Μalnutrition”, and “Nutritional Care”. Additionally, we examined the reference lists of relevant articles and conducted hand searches to identify any potentially relevant articles. We focused mainly on the most recent articles, meta-analyses, and systematic reviews conducted in countries with varying socioeconomic statuses in order to identify possible differential influences.

This narrative review included studies involving adult patients diagnosed with HIV, regardless of gender or ethnicity. Both individuals receiving ART and those not undergoing ART were considered. The review focused on studies assessing nutritional interventions, including dietary modifications, nutritional supplementation, and nutritional counselling. We excluded studies involving children or adolescents under 18 years of age. Additionally, case reports, case series, letters, editorials, and review articles lacking original data were excluded to ensure the inclusion of robust and relevant primary research.

## 3. Results

### 3.1. Pathophysiology of Malnutrition in HIV Disease

Worldwide malnutrition is considered to represent one of the main causes of immunodeficiency [15]. Malnutrition is defined as “the cellular imbalance between supply of nutrients and energy and the body’s demand for them to ensure growth, maintenance, and specific functions” [16].

Malnutrition is strongly associated with a dysregulation of the immune system, thereby potentially promoting increased susceptibility to infections. Dysfunctions of the immune system related to malnutrition are known as nutritional-acquired immune deficiency syndrome (NAIDS). Protein–energy malnutrition (PEM) is characterized by an energy deficit attributed to long-term macronutrient deficiency [17]. The pathogenesis of PEM is multifactorial and associated with adverse effects of HIV infection, resulting in changes to energy intake, nutrient absorption, and/or energy expenditure, ultimately culminating in a negative energy balance (Table 1) [17].

Studies on PEM have demonstrated that a compromised nutritional status has an impact on several aspects of immunity, more particularly on non-specific immunity and elements of specific immunity, such as functions of the T- and B-lymphocytes [18]. Importantly, PEM can cause widespread atrophy of lymphoid tissues, particularly in T-lymphocyte areas in children. Consequently, the thymus gland involutes, leading to a reduction in thymus-derived lymphocyte growth and maturation factors, causing an arrest in lymphocyte development [17]. Furthermore, PEM is associated with reduced levels of mature CD4+ helper cells and impaired antibody production for T-dependent antigens, despite the fact that the counts of CD8+ suppressor cells are relatively preserved [17]. Both HIV infection and PEM may cause a generalized pro-inflammatory reaction, especially in the mucosal barriers, resulting in increased vulnerability to environmental pathogens and opportunistic infections [19]. Although B-lymphocyte counts and functions are usually preserved, immunoglobulin levels, including secretory Immunoglobulin A (sIgA), which is responsible for mucosal immunity, decline. This is attributed to either elevated bacterial adhesion to nasopharyngeal and buccal epithelial cells or modified expression of membrane glycoprotein receptors [17]. Furthermore, severe malnutrition changes the appropriate response of T lymphocytes to interleukin-1 (IL-1), without affecting the synthesis of this monokine itself. In a catabolic state, IL-1 is released by leukocytes inducing endocrine alterations that result in amino acid mobilization, mainly from skeletal muscle. These amino acids are utilized for liver gluconeogenesis and the consequent nitrogen release is excreted in urine [20].

In comparison to a well-nourished state, malnutrition leads to diminished T-cell proliferative responses, lower expression of activation and memory markers on T-cells, and increased polarization of T-helper cells type 2 (T_H_2). This is accompanied by a reduction in the interferon γ (IFN-γ) and interleukin 2 (IL-2) produced by T_H_1 cells [21].

Despite the scarcity of data from studies on PLHIV with concomitant malnutrition, it appears that malnutrition itself is implicated in the suppression of antigen-specific immunity, characterized by decreased T-cell production, maturation, proliferation, and cytokine expression [21]. It is important to note that most findings have been derived from studies including children or adolescents <18 years old; therefore, caution should be exercised when extrapolating conclusions to the adult population.

**Table 1:** Pathophysiology of malnutrition in HIV infection.

Etiology	Mechanisms
HIV-associated wasting	≥10% unintentional weight loss with symptoms for ≥30 days [6] Decreased intake, altered metabolism, inflammatory cytokines (IL-1, IL-6, TNF-α) and increased resting metabolic rate (RMR) [22] TNF-α and IFN-γ contribute to muscle catabolism and oxidative stress [23]
Inadequate dietary intake	Appetite loss due to medications, infections, mouth sores, taste changes, and psychiatric conditions [6] Food insecurity [24]
Nutrient malabsorption	Lean body mass loss, fat malabsorption, and diarrhea due to opportunistic infections (*Cryptosporidium*, *Microsporidia*, *Isospora belli*, *Cyclospora*) lead to chronic malabsorption ART-related diarrhea [25]
HIV enteropathy	Altered gut flora (dysbiosis) impairs intestinal barrier and mucosal immunity, contributing to microbial translocation and chronic immune activation [26] Increased permeability leads to diarrhea, weight loss, and nutrient malabsorption Malabsorption of vitamin B12, bile acid, and monosaccharides occur due to GI structural abnormalities
HIV endocrinopathies	Hypogonadism [27] Adrenal insufficiency or functional adrenal insufficiency due to chronic inflammation (IL-1, IL-6, TNF-α) Glucocorticoid resistance [28,29]

#### 3.1.1. HIV-Associated Wasting

In the pre-ART era, HIV-associated wasting was a common manifestation of HIV infection and was included as an acquired immune deficiency syndrome (AIDS)-defining criterion in 1987 by the Centers for Disease Control and Prevention (CDC) [30]. HIV-associated wasting is defined as an unintentional weight loss ≥ 10% of the person’s initial weight, associated with diarrhea, fever, or weakness for a duration of ≥30 days without a concomitant illness [6]. Despite the widespread use of ART, the incidence of HIV-associated wasting was not eliminated. According to recent guidelines, recommended criteria for HIV-associated wasting include unintentional weight loss of 10% over 12 months or 7.5% over 6 months, or loss of body cell mass of >5% in 6 months [31]. Furthermore, in the literature the term *cachexia* is often substituted for the term *wasting* and it refers to “the combination of anorexia, tissue wasting, weight loss and poor performance status of multifactorial origin” [32].

It is noteworthy that HIV-associated wasting and, notably, the significant loss of metabolically active lean tissue correlates with higher morbidity and mortality [33]. According to the results of the Nutrition for Healthy Living (NFHL) study in Boston, MA, USA, regarding weight loss during the HAART era, the total prevalence of HIV-related wasting was found to be 38%.

Importantly, it was documented that for every 1% loss of body weight from baseline, the risk of death significantly increases [34]. In a recent study by Siddiqui et al. including 42,587 PLHIV, the cumulative HIV-associated wasting prevalence was found to be 18.3% (17.9% on ART, 19.1% not on ART) [35]. Changes in body cell mass (BCM), which represents the intracellular mass of metabolically active tissues of the body, is a more sensitive key tool for the assessment and detection of HIV wasting. It has also been documented that people with HIV demonstrate a progressive depletion of BCM near death [36].

The pathophysiology of HIV-associated wasting is complex and not yet fully comprehended. The main proposed mechanisms include decreased nutrient intake and altered nutrient metabolism under the influence of socioeconomic status, impaired access to care, and medical complications. It appears that numerous interactions have an impact on protein turnover, an integral component of lean mass maintenance. It has been postulated that pro-inflammatory cytokines such as interleukin 1 (IL-1), IL-6 and tumour necrosis factor alpha (TNF-α) are significantly higher in HIV-positive individuals in comparison to HIV-negative individuals. These cytokines lead to a rise in total energy expenditure (TEE) by elevating the resting metabolic rate (RMR) or resting energy expenditure (REE) [22]. Specifically, RMR can rise by 10–30%, particularly during infections, high viraemia, and enhanced protein catabolism [34]. Mallacan et al. concluded that weight loss was attributed primarily to lower energy intake and not to higher energy expenditure [37]. Data from the NFHL cohort revealed that TNF-α and IL-1β levels from stimulated peripheral blood mononuclear cells (PBMCs) were independent predicting factors of lean body mass (LBM) loss and changes in REE [38]. Furthermore, research indicated that nutritional and metabolic abnormalities were more strongly linked to cytokines derived from PBMCs than to plasma cytokines. Consequently, TNF-α, IL-1β, and IL-6 levels in PBMCs were more effective in identifying individuals with HIV-associated wasting based on the CDC definition [34,39].

Among multiple cytokines, TNF-α has also been associated with skeletal muscle catabolism due to inhibition of testosterone secretion, which regulates muscle mass, influences tissue sensitivity to hormone signalling, and directly stimulates protein degradation [40]. Elevated levels of IL-6 and TNF have been associated with higher HIV viral loads. On the other hand, TNF-α and interferon γ (IFN-γ) have been found to suppress myosin expression in muscle cells and therefore lead to anorexia [23]. Additionally, increased cytokine levels may result in chronic oxidative stress in HIV-positive individuals and consequently hasten the course of the disease by affecting immune function, inducing HIV replication, or both [23].

#### 3.1.2. Inadequate Dietary Intake

Dietary intake in individuals with HIV is generally decreased even in the earlier stages of the disease, but this tendency is more prevalent among patients with severe, chronic disease complications. Insufficient energy intake is often attributed to loss of appetite due to medications, mouth sores, impaired taste, or dysphagia [6]. Moreover, neurological or severe psychiatric disease affecting the perception of hunger; nausea; and systemic infections are common causes of reduced dietary intake. It is noteworthy that food insecurity, especially in resource-poor countries, contributes significantly to inadequate energy intake and the inability to meet increased energy needs, as well as inefficient utilization of nutrients [24].

#### 3.1.3. Nutrient Malabsorption

Nutrient malabsorption constitutes a common manifestation among PLHIV presenting clinically as wasting (reduction in lean body mass), fat malabsorption, and diarrhea [41]. Although the extensive use of HAART has resulted in a lower incidence of diarrhea due to opportunistic infections, it is still a major concern in HIV disease management. Particularly, in a cohort study, it was found that fat malabsorption was present in 12.8% of the participants. Poles et al. suggested that high incidence of fat malabsorption (41%) was found while patients were receiving HAART and this may be due to excess growth of small bowel bacteria. However, this finding has not been confirmed by other researchers in similar HIV populations [41,42].

The small intestine is affected in HIV disease either due to opportunistic infections, or to the HIV itself directly leading to malabsorption of most nutrients [43]. Globally, *Cryptosporidium parvum* is a common cause of chronic diarrhea due to protozoa in PLHIV. Microsporidia and, less frequently, *Isospora belli* and *Cyclospora* are also known causes of gastrointestinal opportunistic infections and subsequent chronic malabsorption; they are more common in patients with CD4+ cell counts less than 180 cells/mm^3^ [44,45].

In the ART era, the incidence of infectious diarrhea has decreased, and antiretroviral-induced diarrhea is the most prevalent cause of non-infectious diarrheal disease in PLHIV. The occurrence of this condition varies depending on the specific ART regimen employed, with the highest incidence typically observed in patients undergoing treatment with pharmacologically boosted protease inhibitors (PIs). One survey including 953 people with HIV who were receiving ART demonstrated that approximately 60% reported having diarrhea [25]. Clinical trials data suggest that ART may have resulted in adverse effects in up to 19% of these events.

The pathophysiology of ART-associated diarrhea is unclear. Findings from mouse models showed that ART regimens, including protease inhibitors (PIs) and reverse transcriptase inhibitors, considerably enhanced water and electrolyte secretion into the intestinal lumen in vivo. Rufo et al. showed that PIs, especially nelfinavir, enhance signalling via muscarinic- and calcium-dependent receptors in intestinal cells, resulting in higher chloride secretion into the lumen and ultimately causing secretory diarrhea [46]. An in vitro study by Bode et al. revealed that PIs are also associated with apoptosis in the human intestinal epithelium, resulting in impaired barrier function and increased water secretion into the gut lumen [47].

#### 3.1.4. HIV Enteropathy

HIV enteropathy is an idiopathic type of diarrhea that may manifest at any stage of HIV disease, even without an infectious agent, and is characterized by distinct histologic features [48]. The balance of intestinal flora in the gut is crucial for maintaining a healthy intestinal lining and immune response. When this balance is disrupted (dysbiosis), the gut becomes more permeable, allowing microbes to translocate and trigger chronic immune activation [26]. The exact mechanism of HIV enteropathy is poorly understood, but it is considered to be related to the HIV itself, as well as to the immune system’s response to the virus. It has been postulated that HIV alters the signalling and cellular structure of the gastrointestinal (GI) tract, leading to architectural distortion. HIV enteropathy is characterized by increased intestinal permeability, subsequent diarrhea, and weight loss. Furthermore, these structural abnormalities occurring within the GI tract are associated with malabsorption of vitamin B12, bile acid, and monosaccharides. The histologic features of HIV enteropathy are related to inflammatory lymphocyte infiltrates, with common findings such as villus atrophy, crypt hyperplasia, and villous blunting resulting in diarrhea and malabsorption [47].

#### 3.1.5. HIV Endocrinopathies

Diverse endocrine and metabolic disorders develop due to the direct detrimental impact of HIV, malignancies, and opportunistic infections on endocrine glands, as well as ART-associated toxicities. Hypogonadism is commonly found in men living with HIV and may be related to various manifestations, including fatigue, weight and muscle loss, low libido, and decreased bone density [27]. In the ART era, the prevalence of hypogonadism ranges between 13 and 40% subject to the diagnostic criteria and the participants’ age [49]. A recent systematic review of 41 studies found that the prevalence of testosterone deficiency in males with HIV was 26%. This trend was particularly pronounced when assessing free testosterone levels [50]. Moreover, it was found that hypogonadism prevalence increases with older age, regardless of HIV serostatus [51].

Clinical presentation of adrenal insufficiency with symptoms of weakness and weight loss is rarely observed in HIV patients, especially in the ART era. Infections and malignancies like Kaposi sarcoma and lymphoma in the adrenal and pituitary glands are not common in patients with HIV, though they may be observed in patients who are not receiving ART and in those with antiretroviral drug-resistant infections [52]. Nevertheless, patients with HIV tend to have functional adrenal insufficiency, as they usually have normal or even elevated basal cortisol levels, likely due to a stress response; however, this may be confounded by an increase in cortisol-binding globulin (CBG). Furthermore, adrenocorticotropic hormone (ACTH) levels are usually found to be normal or elevated in patients with increased cortisol levels. These alterations of the hypothalamic–pituitary–adrenal (HPA) axis are attributed to be mediated by cytokines such as IL-1, IL-6, and TNF-α in the context of chronic inflammation.

Acquired glucocorticoid resistance has also been observed in PLHIV, who exhibit relatively elevated baseline cortisol levels compared to HIV-negative controls. Certain medications can also lead to cortisol insufficiency, including ketoconazole, rifampin, megestrol acetate, and opiates [28,29].

### 3.2. Micronutrient Deficiency in Adult HIV Patients

Micronutrients play an essential role in maintaining proper immunologic function and prevent oxidative stress [23]. Vitamins and minerals play a crucial role in the interaction between HIV and nutrition, given their essential functions in cellular differentiation, immune system response, and enzymatic processes. Furthermore, depletion of antioxidant micronutrients may lead to oxidative stress and increase the rate of HIV replication [17].

Single and/or multiple micronutrient deficiencies are prevalent among PLHIV, especially in resource-limited settings, due to insufficient dietary intake and low circulating micronutrient levels. Several studies have postulated that a significant percentage of people with HIV have a lower micronutrients intake in comparison to the Recommended Dietary Allowances (RDAs) for various micronutrients [6].

Shivakoti et al. demonstrated that in a sub-cohort of 270 participants in the PEARLS clinical trial, the prevalence of deficiencies in 0, 1, 2, and multiple micronutrients was 13.9%, 29.2%, 24.5% and 32.4%, respectively. Moreover, in an assessment of how micronutrient status changed after 48 weeks post-ART initiation, it was found that only the mean concentration of vitamin A increased (*p* < 0.001), leading to a 9% decrease in the specific micronutrient deficiency. Consequently, these findings suggest that ART is not sufficient to address micronutrient deficiencies [53]. Another study conducted in Addis Ababa, Ethiopia, including 153 PLHIV, revealed low levels of serum zinc (<10.7 μmol/L) in 53% of participants, serum retinol < 30 μg/dL in 47% of participants, and hemoglobin < 12 g/dL in 4.72% of the subjects [54].

In this context, it is important to review the evidence concerning the impact of essential minerals, trace elements, and vitamins on HIV progression and elucidate the importance of nutrition status assessment and support. Data deriving from nutrition intervention studies provide a significant insight into the role of micronutrients as treatment adjuncts to meet the increased nutrient needs of PLHIV. Various studies have examined the impact of micronutrient dietary supplementation in order to provide nutritional support for PLHIV. The following table (Table 2) summarizes key intervention studies on main trace element supplementation, highlighting their methodologies, nutritional components, and significant findings.

#### 3.2.1. Trace Elements

##### Selenium (Se)

Selenium is an essential micronutrient necessary for the biosynthesis of selenoenzymes like glutathione peroxidase, through incorporation in selenocysteine (Sec). Selenoenzymes are associated with the regulation of oxidative stress in almost all cells, and it was demonstrated that they have a critical impact in both cell-mediated and humoral immune response [58,70].

Data from the literature indicate that the prevalence of selenium deficiency in PLHIV varies between 53% and 71.4%. Several studies have reported an association between HIV infection and lower blood selenium concentrations, highlighting a significant link between HIV disease progression and selenium levels [71]. According to a cross-sectional study of 104 people with HIV at different stages of disease, conducted by Look et al. (1997), mean selenium levels were significantly higher in healthy subjects and CDC HIV stage I patients compared to those in patients in HIV stage II and III [72]. Moreover, Baum et al. reported that selenium deficiency was notably linked to mortality, irrespective of CD4+ cell count, underscoring its independent impact on survival [70].

Several studies examined whether the existing evidence suggests that selenium supplements may be beneficial for people with HIV. A recent systematic review by Muzembo et al. (2019) assessing the impact of selenium supplementation in delaying HIV-infection progression found that daily supplementation with 200 μg of selenium might help maintain the number of CD4+ cells, potentially delaying the progression to AIDS. However, due to heterogenous data from randomized controlled trials (RCTs), there is a need for further investigation regarding the potential of daily selenium supplementation to suppress or reduce HIV viral load in PLHIV [58]. Moreover, Kupka et al. (2008) examined the impact of selenium supplementation in a study of 915 HIV-positive pregnant women in Tanzania. Patients received selenium or a placebo from the 12th to the 27th week of pregnancy, and this treatment continued until six months after childbirth. The results have shown that selenium supplementation reduced morbidity due to diarrhea but had no effect on hemoglobin levels or maternal outcomes [6]. One proposed mechanism underlying the beneficial effects of selenium supplementation involves protection against oxidative stress via glutathione peroxidase, alongside modulation of both cell-mediated and humoral immunity [73]. Furthermore, sufficient selenium levels may boost the production of interleukin 2 (IL-2) and help naïve CD4+ T-cells to proliferate and mature into T helper (Th) 1 cells, thereby improving the cellular immune response against HIV [55]. In contrast, low selenium levels boost immune cells by turning naïve CD4+ into Th2 and, consequently, induce the progression of HIV infection [73]. This discovery may be attributed to HIV’s ability to incorporate host selenium into viral selenoproteins, which serve dual functions as integral components of both glutathione peroxidase and thioredoxin reductase, thereby contributing to selenium deficiency in affected individuals [74]. Another possible mechanism is that selenium may have a pivotal impact regarding the reduction in oxidative stress in HIV-infected cells, potentially suppressing HIV replication rates and consequently becoming depleted [74].

##### Zinc

Zinc is an essential trace element, which is critical for multiple immune functions. Notably, zinc is a structural component of over 750 zinc finger transcription factors, facilitating gene transcription, while it also serves as a catalytic element in approximately 2000 enzymes, including all six classes (hydrolase, transferase, oxido-reductase, ligase, lyase, and isomerase). Thus, zinc plays an integral role in multiple cellular functions, including DNA synthesis and RNA transcription [75], as well as in T-cell proliferation and cytolytic activity [70].

It has been reported that zinc deficiency, as documented by low serum zinc concentrations and/or inadequate dietary zinc intake, is common during several infections, including HIV [18]. Notably, significantly lower serum zinc levels were found in patients infected with both HIV and tuberculosis (TB) in comparison to those in TB patients [76]. Zinc deficiency prevalence among PLHIV varies from 40% to 77% [71]. A retrospective cohort study of 124 men with HIV receiving ART reported levels of serum zinc deficiency in 29.3% of participants, although 58% of the participants had suboptimal zinc intake [71].

Zinc deficiency in PLHIV is linked with inflammation, immunological failure, and increased mortality [61]. Furthermore, a recent study by Martinez et al. (2017) involving a cohort of 487 mono-infected and HIV-HCV co-infected patients found that lower plasma zinc levels were associated with elevated mitochondrial oxidative stress and faster development of liver fibrosis [77]. Poudel et al. investigated the potential role of zinc in relation to inflammatory markers among patients with HIV who were undergoing HAART, reporting that increased serum zinc concentrations were linked to reduced inflammation, as documented by levels of serum C-reactive protein (CRP); this association, however, did not reach statistical significance [78]. Regarding the effect of ART on the zinc status of PLHIV, it was found to be comparable among ART subjects and ART-naïve subjects, whereas it seems to improve serum selenium levels due to a reduction in viral load, which in turn reduces the host’s nutrient requirements [74].

The impact of zinc supplementation in people with HIV was evaluated by numerous studies with inconsistent results. Baum et al. (2010) found that supplementing zinc over a period of 18 months was associated with a fourfold decrease in the risk of immunological failure (number of CD4+ cells < 200 cells/mm^3^) and a 60% reduction in the prevalence of diarrhea (odds ratio, 0.4; 95% confidence interval, 0.183–0.981) in comparison with placebo. Nevertheless, no significant difference in mortality was reported between the two study groups [61]. Furthermore, a recent study by Silva et al. (2021) examining the immunomodulatory role of zinc supplementation in preventing immune failure in HIV-patients with immunovirological discordance (IVD) found that the difference in CD4+ counts between cases and controls was not statistically significant [62]. A Cochrane review by Visser et al. (2017) suggested that, while multiple studies have shown that zinc supplementation had favourable effect, most of them were statistically underpowered. Therefore, the authors came to the conclusion that zinc supplementation may result in increases in blood zinc levels (moderate certainty) and higher numbers of CD4+ cells (low certainty) [59]. In a recent study of 254 ART-naïve patients with a history of heavy alcohol consumption, zinc supplementation was not found to decrease cardiovascular disease and mortality risk, the number of CD4+ cells, inflammation levels, or microbial translocation [63].

##### Copper (Cu)

Copper is an essential micronutrient which plays a crucial role in processes related to energy production and cellular defence against reactive oxygen species. Furthermore, copper is essential for the effectiveness of superoxide dismutase (SOD), which is an important enzyme within the antioxidant defence system that is responsible for scavenging free radicals [77]. Moreover, copper deficiency reduces neutrophilic response to infections and thereby decreases the total number of neutrophils [79]. Several studies have reported that serum copper level and, in particular, the serum copper/zinc ratio are elevated during HIV disease progression and thus might be useful as markers of disease advancement [18]. Nevertheless, it is evident that copper is an acute-phase reactant; therefore, it has been reported that its serum levels increase in response to infection and inflammation due to elevated levels of the circulating copper protein, ceruloplasmin [80]. Importantly, serum copper and zinc levels are controlled by metallothioneins; when copper levels decrease, zinc levels increase, and vice versa. In a study of men with HIV, conducted by Lai et al., it was reported that a copper/zinc ratio > 1.0 was associated with a higher mortality rate [81]. Likewise, in a recent study by Emokpae et al. a negative correlation between the copper/zinc ratio and CD4+ cell count was found [82]. A recent study conducted in Nigeria by Onwuli et al., which assessed the levels of serum copper, zinc, and selenium in 150 subjects with HIV who were receiving ART and ART-naïve treatment, reported a significant decrease in mean values of zinc, selenium and CD4+ T-cells (*p* = 0.0006; 0.0001; 0.0001), respectively. However, the mean values of serum copper were found to be significantly increased, possibly reflecting the non-specific rise in the concentration of copper binding protein ceruloplasmin [74,83].

##### Iron (Fe)

Iron is an essential trace element related to multiple immune functions. Iron serves as a crucial co-factor in electron transfer reactions, oxygen binding and transport, and regulation of cell differentiation and growth. Importantly, iron is implicated in critical cellular processes like energy production (cytochrome C), biosynthesis (amino acid oxidases, fatty acid desaturases), detoxification (cytochrome P450, catalase), oxygen delivery (hemoglobin), oxygen storage (myoglobin), neurotransmitter synthesis (catecholamines) and host defence (nitric oxide synthase, myeloperoxidase, NADPH oxidase) [84]. Iron metabolism is regulated mainly by the hormone hepcidin which is secreted by hepatocytes and binds to ferroportin, the transmembrane protein responsible for transporting iron efflux across the cell membrane [85]. Hepcidin regulates both the intestinal iron absorption and iron release from macrophages in the reticuloendothelial system [86].

The association between iron status and HIV infection is intricate and bidirectional. Iron deficiency has been linked to compromised immune function, reduced macrophage bactericidal activity, and impaired T-cell function [87]. On the other hand, higher cellular iron levels have been associated with increased HIV infection and replication [88]. Chang et al. have demonstrated that HIV infection alone results in increased cellular and systematic iron levels, regardless of treatment with ART [88]. Likewise, several ART drugs were found to have a direct effect on iron homeostasis, leading to increased cellular iron, independently of HIV infection [89]. Additionally, it has been reported that while iron stores may decrease during early asymptomatic HIV infection, possibly due to compromised absorption, they may increase as the disease progresses, leading to iron accumulation in macrophages and other cells [89].

Anemia is a frequent health problem among PLHIV, and it is associated with HIV mortality and disease progression [90,91]. The overall prevalence of anemia among adults with HIV was estimated at 46.6% (95% CI: 41.9−51.4%) [92]. In the course of HIV infection, anemia is often related to iron deficiency (ID), anemia of chronic disease, coexisting infections, or represents an adverse effect of the antiretroviral therapy (ART) [67]. A recent study of 40,657 adults on ART who were living in Tanzania revealed that the association between mild, moderate, and severe anemia at ART initiation with the risk of all-cause mortality was found to be 1.26, 1.90, and 3.32, respectively, indicating that the gravity of anemia substantially increased the risk of death [66]. In concordance with these findings, a recent systematic review by Abioye et al. found that anemia is linked to an increased risk of all-cause mortality and the occurrence of tuberculosis in PLHIV, regardless of its severity or etiology [67]. Furthermore, elevated serum ferritin is also correlated with detrimental clinical outcomes, although it has not been determined whether this is the result of high iron concentrations or inflammation due to disease advancement [67]. Data from longitudinal studies regarding the significance of iron status for HIV-related health outcomes showed that treatment with ART leads to the resolution of anemia in adults and pregnant women [93,94]. In a recent study conducted by Garrido-Rodríguez et al. (2022), the iron status of a cohort including virologically suppressed chronic people with HIV receiving ART was assessed. The study found altered iron metabolism regulators, indicating a complex functional iron deficiency [85].

Iron supplementation in PLHIV poses a complex challenge. While it can improve hemoglobin levels and reduce anemia persistence, concerns exist with regard to its safety and potential adverse effects [67]. Although low-dose iron supplementation does not seem to increase HIV-1 virus load, data from observational studies suggest that it may increase HIV replication and accelerate disease progression [65]. The association between iron status and HIV outcomes is further complicated by the impact of inflammation and the consequent difficulty in distinguishing between iron deficiency and anemia of chronic disease [67]. In a randomized controlled trial with HIV and female injected-drug users with hepatitis C, it was found that supplementation with daily micronutrients, including iron, can reduce anemia and improve iron status, without elevating plasma HCV or HIV RNA levels [64]. However, one large observational study in Tanzania found an increased mortality risk in adults with HIV who received iron supplementation, regardless of the severity of anemia [66]. Moreover, a recent observational study with patients initiating HAART reported that multiple micronutrient supplements (MMSs) can also reduce anemia and mortality risk [68]. The above-mentioned results indicate that MMS could be a substitute for iron supplementation, since data related to the safety and efficacy of isolated iron supplementation raise concerns. Nevertheless, the appropriate dosage of iron in MMS used in PLHIV is has yet to be documented [67]. Importantly, iron supplementation has been found to have different impacts on mortality and viral load depending on whether the patients with HIV are initiating or continuing HAART. Thus, an increased risk of unsuppressed viral load in PLHIV continuing, but not initiating, HAART and the stronger negative association in PLHIV with Stage III/IV disease in comparison with Stage I/II disease has been documented [69].

In conclusion, results from observational studies have variable degrees of adjustment for confounding variables; thus, large-scale, randomized trials investigating courses of safe and effective iron supplementation among PLHIV are warranted.

#### 3.2.2. Vitamins and HIV Infection

Vitamins have a pivotal impact on the function of the immune system and on overall health, making them particularly important in relation to HIV infection. Numerous studies indicate that vitamin deficiencies are prevalent among people with HIV and may contribute to disease advancement and transmission. Insufficient levels of vitamins A, E, B6, B12, and C have been associated with higher mortality rates by increasing oxidative stress and compromising immunity [95]. These deficits may arise due to inadequate intake, malabsorption, and metabolic disturbances [95]. Thus, vitamin supplementation may have a favourable effect on epithelial integrity and systemic immunity, decreasing viral load and enhancing clinical outcomes [96]. However, the impact of supplementation differs depending on the specific vitamin. Studies highlighting the impact of the main vitamins A and D on viral load and other immunological variables in adults with HIV are summarized in Table 3.

Vitamin A includes a number of retinoid compounds exhibiting the biological activity of all-trans-retinol [97]. Vitamin A and its related retinoids are pivotal in regulating both innate and cell-mediated immunity. They are instrumental in maintaining epithelial tissue integrity in the respiratory and gastrointestinal tracts, while also preventing conditions such as night blindness [98]. Additionally, evidence suggests that vitamin A increases natural killer cell function in vitro and stimulates phagocytosis [99]. Importantly, β-carotene, a precursor of vitamin A, has been found to contribute to the maintenance of the immune response and may also act as an antioxidant [100].

It is well documented from cross-sectional studies that vitamin A deficiency is prevalent in PLHIV compared to healthy populations [101,102]. A 2017 Cochrane review by Wiysonge et al. evaluated the impact of vitamin A supplementation among HIV-positive women during pregnancy and after childbirth. This study reported that vitamin A supplementation probably had minimal or no effect on mother-to-child transmission (MTCT) of HIV (RR 1.07, 95% CI 0.91 to 1.26; 4428 women, five trials, moderate-certainty evidence) and may have minimal or no effect on child death by two years of age (RR 1.06, 95% CI 0.92 to 1.22; 3883 women, three trials, low-certainty evidence). However, evidence suggests that the use of vitamin A supplements during pregnancy may lead to an increase in mean birthweight (low-certainty evidence) and is likely to decrease the incidence of low birthweight (moderate-certainty evidence) [103].

Vitamin D is a steroid hormone synthesized from a cholesterol precursor (7-dehydrocholesterol) under the influence of sunlight (UVB 290–315 nm), forming pre-vitamin D3. Consequently, pre-vitamin D3 undergoes thermal isomerization to vitamin D3. Further metabolism of vitamin D to its major circulating forms, 25(OH)D and 1,25(OH)D, takes place in the liver and kidneys, respectively, as well as in other tissues, where the 1,25(OH)2D serves a paracrine/autocrine function, such as in the skin, immune system cells, parathyroid gland, intestinal epithelium, prostate, and breast [104]. Vitamin D3 is carried in the bloodstream and is often bound to vitamin D-binding protein (DBP), an α-globulin produced in the liver. The main forms of vitamin D in humans are vitamin D2 or ergocalciferol, synthesized from ergosterol in plants, and vitamin D3 (cholecalciferol), synthetized naturally from cholesterol in animals. Most functions of 1,25(OH)2D are mediated by the vitamin D receptor (VDR) [105].

In addition to the major role of vitamin D in bone metabolism, evidence has demonstrated its immunomodulatory effect, as VDR is found to be expressed in T and B cells, dendritic cells, and macrophages. Furthermore, vitamin D controls the production of antimicrobial cytokines, such as TNF-α and INF-γ, and induces the production of antimicrobial peptides, like cathelicidin and defensins [70,106]. Notably, it was found that reduced concentrations of vitamin D below 20 ng/mL were associated with a suppressed cathelicidin response and a subsequent increase in opportunistic infections [107]. The criteria for vitamin D deficiency and insufficiency in adults are defined as total 25(OH)D concentrations < 25 nmol/L (10 ng/mL) and <75 nmol/L (30 ng/mL), respectively.

A considerable number of studies assessed vitamin D status among PLHIV. A recent systematic review by Wang et al. demonstrated a modest but significant higher vitamin D deficiency prevalence in PLHIV than in control subjects (odds ratio of 1.502 (95% CI, 1.023–2.205; *p* = 0.038). Furthermore, it was indicated that HIV-individuals were prone to vitamin D deficiency when they were receiving ART, living in lower latitudes, of advanced age, had a lower BMI, and were male [108]. In a recent review, Mansueto et al. estimated the high overall prevalence of 25(OH)D deficiency among PLHIV, ranging from 70.3 to 83.7% based on a large number of epidemiological studies with varying thresholds [109].

The potential role of vitamin D supplementation in people with HIV was evaluated in a recent review of 29 clinical studies by Alvarez et al. [110]. The evidence suggested that regardless of ART use, when vitamin D concentrations were normal, a reduction in inflammation markers was noticed; this was correlated with reduced bone turnover and decreased risk of secondary hyperparathyroidism, and with an increased antibacterial response. Moreover, three out of seven studies assessing the impact of vitamin D supplementation on immunologic parameters reported elevated numbers of CD4+ T cells, whereas there was no clear evidence regarding its impact on viral load, since most patients were receiving ART [110].

**Table 3:** Vitamin A and D supplementation studies in HIV-positive individuals.

Study	Study Design	Sample Size	Assessment Tool	Nutritional Intervention	Study Results
Semba et al. (1998) [98]	Double-blinded RCT	N = 120 HIV-infected injection drug users	CD4+ count viral load	60 mg retinol equivalent (200,000 IU) for 4 weeks	Vitamin A supplementation had no significant impact on HIV load or CD4+ lymphocyte count at 2 and 4 weeks after treatment
Wiysonge et al. (2017) [103]	Cochrane Database of Systematic Reviews	N = 4428	Risk of MTCT	Vitamin A supplements in HIV-positive women during pregnancy, during the immediate postpartum period, or both	There was no overall evidence that vitamin A supplementation reduced MTCT of HIV. Vitamin A significantly improved birth weight but did not affect other maternal and infant outcomes like stillbirths, preterm births, infant mortality, maternal CD4+ levels, and maternal mortality. One trial in Tanzania showed that vitamin A supplementation increased the risk of mother-to-child transmission of HIV.
Lachman et al. (2015) [111]	Pilot, open-label, three-arm prospective phase 1 study.	N = 17 HIV-positive patients	T-cell flow cytometry methods	A single dose of 200,000 IU oral cholecalciferol for 4 weeks	Vitamin D supplementation led to increased frequencies of antigen-specific T cells expressing the anti-HIV chemokine MIP-1β, which correlated with the rise in vitamin D levels
Alvarez et al. (2019) [110]	Review (29 clinical studies)			Oral vitamin D3 (cholecalciferol) supplementation, with one study also using intramuscular supplementation. Daily dose ranged from approximately 400 to 14,000 IU (4000 and 7000 IU as the most common doses) from 4 to 104 weeks.	Vitamin D supplementation can decrease inflammation, bone turnover markers, and the risk of secondary hyperparathyroidism, while increasing the antibacterial response. In 3 out of 7 studies, vitamin D supplementation led to an increase in CD4+ T cell count, although its effect on viral load was inconclusive, since most patients were on HAART.

Mother-to-child transmission (MTCT).

## 4. Discussion

Malnutrition presents a multifaceted and complex challenge on a global scale among PLHIV, intensifying the severity of the effects of the virus. This dynamic can underlie a vicious cycle wherein the compromised immune system amplifies nutritional deficiencies. PLHIV frequently experience deficiencies in essential trace minerals and vitamins which are linked to accelerated disease progression and increased susceptibility to opportunistic infections. While ART has significantly improved the prognosis and quality of life of a great many people with HIV, malnutrition remains a major public health concern, especially in resource-poor settings. This comprehensive analysis of the recent literature highlights the intricate relationship between altered nutritional status and the progression of HIV infection. Numerous studies have demonstrated the high prevalence of specific micronutrient deficiencies in PLHIV, with multiple implications for immune function, disease progression, treatment outcomes, and an associated increase in the morbidity and mortality of this population.

Additionally, this review highlights the importance of integrated care models that incorporate nutritional interventions into the overall management of HIV, aiming to enhance micronutrient status and improve the well-being of PLHIV. Multiple nutritional intervention studies have evaluated the role of micronutrients in the management of HIV infection and improving clinical outcomes, with particular emphasis on selenium, zinc, copper, iron, and vitamins as critical components. These nutrients have an impact on oxidative stress, immunological response, and metabolic homeostasis, highlighting their potential beneficial role as adjuvant therapies.

Specifically, findings from clinical trials have demonstrated that selenium supplementation may delay CD4+ cell count decline and boost the immune system. However, data regarding its impact on viral load and mortality risk remain inconclusive due to heterogeneity in study designs and outcomes. Data supporting the beneficial impact of zinc supplementation are also inconsistent. Although some studies indicated improvement in CD4+ cell count and a reduction in diarrhea rate, others showed limited or no difference in mortality risk. Additionally, the relationship between zinc status, ART, and co-infections with conditions like tuberculosis requires further investigation. Regarding copper, the serum copper/zinc ratio was found to be elevated in several studies, indicating a potential role as a biomarker of HIV progression. Furthermore, copper constitutes an acute-phase reactant, increasing in response to infection and inflammation. However, the exact impact of altered copper levels in HIV disease remains unclear. The dual role of iron as both an essential micronutrient and a potential contributor to increased HIV replication highlights the intricate relationship of this trace element with HIV disease. There is also evidence that ART can alter iron homeostasis, contributing to the higher prevalence of anemia among PLHIV. Thus, given the safety concerns regarding iron supplementation, research should be focused on the development of standardized protocols, particularly in combination with multiple micronutrient supplements (MMSs).

It is noteworthy that both vitamin A and D deficiencies are prevalent in PLHIV, resulting in an impaired immune response. Vitamin A plays a pivotal role in immune regulation and maintenance of epithelial integrity, while vitamin D modulates both innate and adaptive immunity. Although supplementation studies with these vitamins indicate a potential benefit in terms of reducing inflammation and improving CD4+ counts, evidence remains inconsistent in terms of mortality.

This review also provides insight into the challenges of determining the optimal dosage and timing of supplementation and the potential interactions with ART, in the context of various nutritional needs across different stages of HIV infection and demographic factors. The present overview reveals that even though supplementation with single or multiple micronutrients can address micronutrient deficiencies in PLHIV, there is inadequate evidence regarding treatment outcomes. Additionally, the small sample sizes of nutrition intervention RCTs and inconclusive data regarding the safety and effectiveness of micronutrient supplementation underscore the necessity of tailored nutritional interventions.

While the current narrative review provides valuable insights into the role of trace elements and vitamins in HIV management, it is subject to certain limitations that may impact the strength and applicability of its conclusions. As a narrative review rather than a systematic review, there are inherent limitations related to the reproducibility of findings. Furthermore, the literature search was conducted primarily in the English language and might therefore have excluded relevant studies published in other languages, especially in resource-limited settings where HIV prevalence and micronutrient deficiencies are extensive. The significant heterogeneity of the included studies—and particularly the disparities in geography, demographic and socioeconomic status among HIV populations—could be confounding factors in data interpretation. It should also be noted that the narrative review included nutritional intervention studies with varying dosages and treatment durations, making it difficult to identify optimal supplementation strategies or draw consistent conclusions about efficacy and safety. Lastly, the articles in the review focus on the potential benefits of enhancing micronutrient status and supplementation, but the long-term impacts on the mortality and quality of life of PLHIV have not been sufficiently examined.

## 5. Conclusions

In conclusion, this narrative review highlights the complex relationship between malnutrition and HIV, exploring the challenges inherent in the comprehensive management of this disease. As summarized in the literature, the interplay of nutritional status with HIV and its effect on the immune system indicates the need for patient-centred interventions that address both the nutritional and immunological aspects of HIV infection. Further research based on large-scale RCTs is crucial to establish national guidelines and standardized protocols for micronutrient supplementation in PLHIV. Importantly, longitudinal studies assessing the long-term impact of supplementation on mortality and quality of life of PLHIV are imperative, as these will provide robust evidence for the optimal management of HIV disease. To address the impact of undernutrition among individuals with HIV, innovative public health strategies incorporating nutritional education and support should be an integral part of any comprehensive healthcare plan. By addressing the aforementioned gaps in our knowledge, future studies can better define the role of micronutrient supplementation in the holistic care of PLHIV, improving their overall survival and well-being.

## Figures and Tables

**Table 2:** Micronutrient supplementation studies in HIV-positive individuals.

Study	Study Design	Sample Size	Assessment Tool	Nutritional Intervention	Study Results
Kamwesiga et al. (2015) [55]	Double-blind RCT	N = 300 ART-naïve patients with HIV	CD4+ count	Selenomethionine (200) for 24 months	Reduction in monthly rate of CD4 cell depletion
Baum et al. (2013) [56]	Double-blind RCT	N = 878 ART-naïve patients with HIV	CD4+ count, viral load	Supplementation with either daily multivitamins (B vitamins and vitamins C and E), selenium alone, or multivitamins with selenium vs. placebo for 24 months.	Supplementation with a single supplement containing multivitamins and selenium significantly reduced the risk of reaching a CD4 cell count of 250/μL or less and 350/μL or less and was safe and well tolerated.
Hurwitz et al. (2007) [57]	Double-blind RCT	N = 262 patients with HIV receiving ART	CD4+ count, viral load	Selenium yeast supplementation (200 μg/d) for 9 months	Selenium supplementation led to a significant increase in serum selenium levels, which was associated with decreased HIV-1 viral load and increased CD4+ count.
Muzembo et al. (2019) [58]	Systematic review	N = 3642	CD4+ count, viral load	Selenium 200 μg/day for 9–24 months	Selenium supplementation can reduce the decrease in CD4+ counts. Inconclusive results on HIV viral load.
Visser et al. (2017) [59]	Cochrane Database of Systematic Reviews		CD4+ count, viral load, serum selenium levels	Selenium 200 μg/day (9–24 months) Zinc 12–50 mg/day (2 weeks–18 months)	Selenium supplementation has minimal or no impact on CD4+ count or viral load; may lead to an increase in blood selenium levels. Zinc supplementation has minimal or no impact on CD4+ count or viral load; unknown effect on diarrhea frequency.
Kayode et al. (2020) [60]	Systematic Review	N = 1970 (selenium) N = 1535 (zinc)	CD4+ count, viral load, serum selenium levels	Selenium 100–200 μg/day (9–24 months) Zinc 12–50 mg/day (1–18 months)	Selenium supplementation led to an increase in serum selenium levels, CD4+ cell counts and a decrease in HIV viral load; reduction in hospital admissions; decrease in diarrhea morbidity in pregnant women. There is some evidence suggesting that zinc supplementation may have a beneficial effect on reducing diarrhea morbidity and improving immunological function.
Baum et al. (2010) [61]		N = 231 HIV-positive patients	CD4+ count, viral load	Zinc supplementation for 18 months	4-fold decrease in immunological failure (CD4+ < 200 cells/mm^3^); no effect on viral load; reduced HIV-related diarrhea rate by >50% (OR = 0.4, 95% CI: 0.183–0.981, *p* = 0.019); no significant mortality difference between groups.
Siva et al. (2021) [62]	Double-blind RCT	N = 40 IVD HIV-positive patients	CD4+ count, viral load	Zinc supplementation for 12 months	In IVD patients, median CD4+ increase: 31.5 cells/mm^3^ (zinc) vs. 50 cells/mm^3^ (placebo), not statistically significant (*p* = 0.382).
Freiberg et al.(2020) [63]	Double-blind RCT	N = 254 ART-naïve patients with HIV with past 30-day heavy alcohol consumption	Mortality risk assessment (VACS Index score)	Zinc supplementation for 18 months	There was no reduction in mortality risk, CD4+ cell counts, cardiovascular disease risk, and levels of inflammation or microbial translocation.
Semba et al.(2007) [64]	Double-blind RCT	N = 138 HIV-positive female injection drug users infected with hepatitis C.	Log10 plasma RNA (copies/mL)	Daily micronutrients with 18 mg of iron (iron group) vs. micronutrients without iron (control group) for 12 months	A daily micronutrient supplement with iron can reduce anemia and improve iron status in female injected-drug users without increasing plasma HCV or HIV RNA levels or altering liver enzymes.
Olsen et al. (2004) [65]	Double -blind RCT	N = 45 HIV-positive patients, ART-naive	Viral load	60 mg iron (ferrous dextran) twice weekly for 4 months	There was no effect on HIV-1 viral load. Observational data suggest that iron may increase HIV replication and disease progression, which is a concern for efforts to combat iron deficiency.
Haider et al. (2019) [66]	Prospective cohort study	N = 40,657 patients with HIV receiving HAART	Anemia, ID, mortality	Iron supplements (ferrous sulphate 200 mg and folic acid 5 mg) given daily to participants found to be anemic, until their anemia resolved or for up to 6 months	Anemia and iron deficiency anemia are associated with higher mortality risk in HIV patients receiving ART and iron supplementation may increase mortality risk.
Abioye et al. (2020) [67]	Systematic Review		Iron status indicators and clinical outcomes (mortality, TB, CD4+ counts, HIV viral load)		- Anemia, regardless of its etiology, is linked to a higher risk of all-cause mortality and tuberculosis among PLHIV, with the risk increasing as anemia becomes more severe. - Elevated serum ferritin is associated with negative clinical outcomes, though it is uncertain whether this is due to high iron levels or inflammation from disease progression. - A large observational study found an increased risk of mortality in adults with HIV who received iron supplementation.
Sudfeld et al. (2019) [68]	Retrospectivecohort study	N = 67,707 HIV-positive individuals (48,207 ART-naïve)	Mortality risk	Multivitamin supplements (vitamins B-complex, C, and E)	- In ART-naïve patients, multivitamin supplements reduced mortality risk, meeting ART eligibility criteria, developing anemia or TB, progressing to a more advanced WHO HIV disease stage; reduced mortality, incidence of tuberculosis, and immunologic failure among ART-experienced adults. - The mortality benefits of multivitamin supplementation may have been limited to the first year of ART treatment.
Abioye et al. (2024) [69]	Prospective cohort study	N = 70,442 HIV-positive people initiating or continuing HAART	Mortality, viral load	Iron supplementation	Iron supplementation use was associated with reduced mortality risk, but an increased risk of unsuppressed viral load among PLHIV that were continuing HAART. Participants with stage III/IV disease exhibited a higher negative correlation between iron supplement use and the likelihood of having an unsuppressed viral load than those with stage I/II disease.

RCT—randomized control trials; CD4+—cluster of differentiation; IDV—immunovirological discordance; ID—iron deficiency; VACS—veterans ageing cohort study; HAART—highly active antiretroviral therapy; PLHIV—people living with HIV; TB—tuberculosis.
